# Circular RNAs as novel biomarkers with regulatory potency in human diseases

**DOI:** 10.4155/fsoa-2018-0036

**Published:** 2018-05-23

**Authors:** Yuan Fang

**Affiliations:** 1 The MOH Key Laboratory of Geriatrics, National Center of Gerontology, Beijing Hospital, 1 Dahua Road, DongCheng District, Beijing, PR China

**Keywords:** biomarkers, characterization, circRNAs, function, human disease, regulators

## Abstract

Circular RNAs (circRNAs) are a large class of noncoding RNAs characterized with closed loop structures without 3′ and 5′ polar ends. They can roughly be divided into exonic circRNAs, exon–intron circRNAs and circular intronic RNAs. CircRNAs are characterized with stability, prevalence, specificity and conservation, which arouse great interest in circRNAs as disease biomarkers. Their abilities to sponge to miRNAs, cis-regulate parent genes, bind to proteins and encode proteins endow circRNAs a critical role of regulation in eukaryotic cells. This concise review focuses on circRNAs as functional biomarkers and therapeutic targets in both tumor and nontumorous diseases.

## Lay abstract

Although they were discovered in 1970s, circular RNAs (circRNAs) have attracted great interest only relatively recently. Instead of genome ‘junk matters’, circRNAs are now considered as promising biomarkers and treatment targets. CircRNAs are involved in numerous cancer-related and noncancer diseases, such as lung cancer, gastric cancer, cardiovascular diseases, diabetes mellitus and so on. This review outlines the classification, characterization and function of circRNAs, with a specific focus on recent studies concerning the role of circRNAs in human diseases.

Circular RNAs (circRNAs) are a class of endogenous noncoding RNAs characterized with closed ring structure without 3′ and 5′ ends. Although circRNA transcripts were firstly described decades ago [1], they have long been considered as low-level ‘transcriptional noise’ with little or no regulatory potential [2]. This phenomenon may result from traditional RNA detection methods depending on poly-A ends, which unavoidably filter out back-splicing sequences without free ends. In recent years, with the development of deep sequencing and bioinformatic approaches, a large number of circRNAs have been uncovered with stable expression in eukaryotic cells [3,4].

CircRNAs can mainly be divided into three subtypes (Figure 1), namely exonic circRNAs (ecircRNAs) [4], exon–intron circRNAs (EIciRNAs) [5] and circular intronic RNAs (ciRNAs) [6]. EcircRNAs are predominantly localized in the cytoplasm, such as Cdr1as, Sry and circ-HIPK3 [4]. Most ecircRNAs are generated from back-splicing of pre-mRNAs, in which downstream donor-exons splice to upstream acceptor-exons [7]. EIciRNAs are abundant in the nucleus. These circRNAs are formed with introns ‘retained’ between exons during the back-splicing process [5]. CiRNAs, containing two or more connected introns, localize mainly in the nucleus. Their processing depends on consensus motifs containing 7 nt GU-rich elements close to the 5′ splice site and 11 nt C-rich elements proximal to the branch point site [6].

**Figure 1.**
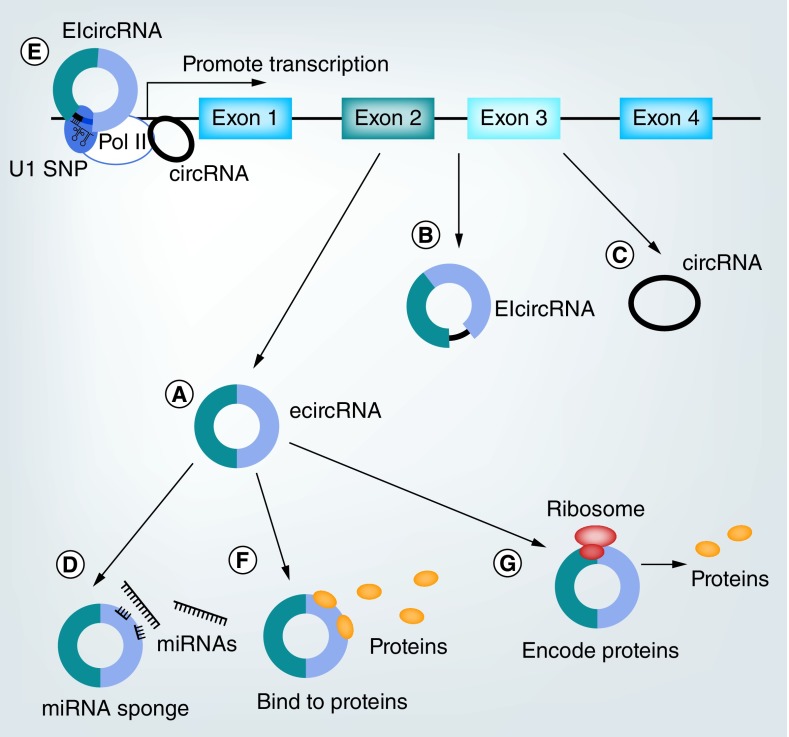
**Regulatory functions of different types of circular RNAs.** **(A–C)** Three types of circular RNAs (circRNAs). **(A)** EcircRNAs are generated from exons. **(B)** EIcircRNAs contain both exons and introns. **(C)** CircRNAs are formed by introns. **(D–G)** Functions of circRNAs. **(D)** CircRNAs can act as miRNA sponges. **(E)** CircRNAs are able to promote transcription. **(F)** CircRNAs can bind to proteins. **(G)** CircRNAs encode proteins. CircRNA: Circular intronic RNA; EcircRNA: Exonic RNA; EIcircRNA: exon–intron RNA.

The expression of circRNAs is stable, prevalent, specific and conserved. Compared with their linear counterparts, circRNAs are more resistant to RNase R-mediated degradation and have longer half-lives in some cases [4]. CircRNAs are abundant in various eukaryotic cells and human tissues, with parts of the circRNAs highly conserved among different species [3,8]. The expression of circRNAs shows spatio-temporal specificity, and changes during the disease process, indicating an interesting function of circRNAs in physiological and pathological conditions [9]. In 2013, the ‘sponging’ function of circRNA was first demonstrated, and it was found that circRNAs can suppress miRNA function by targeting seed sequences [10,11]. Later on, the role of circRNAs as promising noninvasive biomarkers with regulatory potency was investigated in various diseases such as cardiovascular [12], endocrine [13], autoimmune [14] and CNS diseases [15], and cancer [16]. Other functions of circRNAs, such as cis-regulation and protein coding, also suggest the potential role of circRNAs in human disease. This review summarizes the characterization and function of circRNAs and investigates the association between circRNAs and numerous diseases including cancer and noncancer diseases.

## Characteristics

CircRNAs are shown to demonstrate stable, specific and conservative expression patterns.

### Stability & prevalence

CircRNA transcriptions have no free 3′ and 5′ ends, thus are more resistant to RNase R and more stable than corresponding linear RNAs in most cases. In cells, although the circRNAs exhibit average half-lives of more than 48 h, the half-lives of associated linear RNAs are less than 20 h [4]. The stability may lead to a more abundant expression of circRNAs. For example, the abundance of circular transcripts of *HIPK3* is roughly fivefold more than their linear counterparts. In some cases, the circular transcripts are estimated to be tenfold more abundant than cognate linear mRNA [4,9,17].

The prevalence of circRNAs is not unique in mammal cells, but is common in the eukaryotic world, from mammals to insects, plants and fungi [4,10]. In human beings, a large number of circRNAs are detected in diverse clinical specimens, such as whole blood [18], plasma [19] and saliva [20]. It is reported that in human whole blood samples, while circRNA isoforms are already detectable, in hundreds of cases the corresponding linear transcripts are absent [18].

### Specificity

Oftentimes, the expression of circRNAs alters in different cell lines. For instance, the circular ratios of gene *AFAP1, ASPH* and *SH3PXD2A* are significantly different in BJ-T, HEK293 and HeLa cells [7].

CircRNAs also have specific expression in different tissues. In human beings, some circRNAs accumulate more in the CNS, especially synapses, compared with other tissues such as thyroid gland, liver and muscle [9]. Similar expression patterns can be observed in mouse and *Drosophila*. For example, circRNA generated from *Rims2* is highly expressed in adult mouse brain while it can rarely be detected in other mouse tissues [9]. In *Drosophila*, more than 90% of circRNAs identified in any fly tissues can also be detected in the head while half of the circles observed in the head cannot be detected in other tissues [21]. Tissue-specific expression is also observable in plants. For instance, in polyploidy *Gossypium* species, the overall expression level of circRNAs is higher in ovule samples than leaf samples. In addition, more than 80% circRNAs express only in ovule tissues [22].

Moreover, circRNAs are expressed in a developmental-stage specific manner. In mammalian and *Drosophila* brains, the global expression of circRNAs varies in different stages. For instance, mouse circRNAs derived from the *Staufen2* gene exhibit reciprocal expression during neuronal differentiation [9]. In *Drosophila*, circRNA accumulation is associated with the aging process of the brain. The level of brain circRNAs is elevated from embryo to larva and pupa, and is even higher in adult heads [21].

### Conservation

A small proportion of circRNAs is highly conserved across different species. In a research performed by Dong *et al*., the authors found about 15,000 circRNAs in both human and mouse genomes, indicating that 15% of total human circRNAs and 40% of total mouse circRNAs are conserved [23]. In another study concerning circRNA expression in heart, the authors discovered that about 10% cardiac circRNAs are conserved across human, mouse and rat, and about 30% cardiac circRNAs can be found simultaneously in mouse and rat [24]. In a recent study conducted by Stoll *et al*., among the 3441 explored human pancreatic islet circRNAs, 497 orthologous circRNAs can be determined in parallel mouse islet samples [25]. The conserved expression of circRNAs is associated with complementary intronic sequences flanking back-spliced exons [23]. Orthologous circRNAs across species often exhibit longer flanking introns than species-specific ones [22].

### Nomenclature

With the advancement of next generation sequencing and bioinformatic technology, a large number of circRNAs are detected for the first time. Some newly discovered circRNAs are nominated by their founders concerning the type, location or function of circRNAs (Figure 1). For example, ci-ankrd52, a ciRNA generated from parent gene *ANKRD52*, is named after its genome location. The name of a well-known ecircRNA, ciRS-7, contains the meaning of circRNA sponge for miR-7. However, such nomenclature can be confusing because one gene can generate several circular transcripts and one miRNA can become targets for different circRNAs. Luckily, existing databases, such as circBase (http://circbase.org/), allow one to assess the variety of names, forms and functions of circRNAs.

## Functions

Although the exact regulatory mechanism of circRNAs is still unclear, current research concentrates mainly on the following four aspects, namely sponging of miRNAs, cis-regulating of parent genes, binding to proteins and encoding proteins (Figure 1). What is more, the categorization and localization of circRNAs are important to analyze their functions (Table 1).

**Table 1.** **Circular RNAs classification and function.**

**Type**	**Derivation**	**Location**	**Function**	**Examples**	**Ref.**
EcircRNA	Exons	Mainly cytoplasm	MiRNA sponge; cis-regulation at post-transcriptional level; protein binding; encode functional proteins	Cdr1as; circ-ITCH; hsa_circ_0031288; circ-FBXW7	[10,26–28]

EIcircRNA	Exons and introns	Mainly nucleus	Cis-regulation of parent gene	circEIF3J; circPAIP2	[5]

CircRNA	Introns	Mainly nucleus	Cis-regulation of parent gene	circ-ankrd52; circ-mcm5; circ-sirt7	[6]

CircRNA: Circular intronic RNA; EcircRNA: Exonic RNA; EIcircRNA: exon–intron RNA.

### CircRNAs as miRNA sponges

Lines of evidence indicate that natural circRNAs serve as effective miRNA sponges (Figure 1D). MiRNAs are small noncoding RNAs (19–22 nt) which negatively modulate mRNA expression in post-transcriptional stage via binding to 3′-untranslated regions [29]. CircRNAs localized in cytoplasm can interact with both miRNA and AGO, and sequentially eliminate the suppression of miRNAs on mRNA. For example, the first seriously studied circRNA, Cdr1as, harbors more than 70 conserved miR-7 binding site. The effect of Cdr1as expression simulates that of miR-7 silencing, indicating a role of miRNA binding [10,11]. Another well-known circRNA, Sry, can serve as competitive inhibitor for miR-138 by binding to target sites [11]. Although one paper published in 2014 indicated that few circRNAs could act as efficient miRNA sponges [30], more and more circRNAs have been demonstrated in recent years to shape gene expression via inhibiting miRNAs. For example, circHIPK3 contains 18 potential binding sites for nine different miRNAs [31]. CircHIPK3 plays an important role in hepatocellular carcinoma (HCC) by sponging miR-124, leading to abnormal cancer cell proliferation and migration [32]. CircHIPK3 is also able to regulate endothelial proliferation and vascular dysfunction in diabetic retinopathy (DR) via blocking miR-30a-3p [33].

### Cis-regulation of parent genes

CiRNAs are able to regulate the expression of their parent genes in cis (Figure 1E). Nuclear ciRNAs localized near the transcription sites of their parent genes can interact with RNA Pol II elongation machinery and act as positive regulators for transcription. For example, knockdown of ci-ankrd52, ci-mcm5 and ci-sirt7 results in suppressed expression of their parental mRNAs [6]. EIciRNAs are also capable to cis-regulate their parental genes. EIciRNAs are able to promote transcription of RNA Pol II through interacting with U1 snRNP [34]. Silencing of two EIciRNAs, circEIF3J and circPAIP2, reduces the expression level of their parental mRNAs [5].

The majority of ecircRNAs acts as miRNA sponges and does not regulate the expression of their cognate genes. However, circ-ITCH shares the same miRNA response elements with the 3′-untranslated region of *ITCH* mRNA. By binding to miRNAs, circ-ITCH relieves the suppressive effect of miRNA on its own parent mRNA. The enhanced expression of *ITCH* at post-transcriptional level by circ-ITCH finally results in the suppressed activity of Wnt/β-catenin pathway. Dysregulation of circ-ITCH and Wnt/β-catenin pathway are involved in the progression of lung cancer, esophageal squamous cell carcinoma and colorectal cancer (CRC) [26,35,36].

### Protein binding

Except miRNAs, circRNAs are also able to interact with other entities, such as proteins (Figure 1F). In a study conducted by Du *et al*., circ-Foxo3 was demonstrated to influence cell cycle progression by binding to two cell cycle proteins (CDK2 and p21) [37]. PES1 is an important protein essential for ribosome biogenesis. CircANRIL can attach to PES1 and increase apoptosis of cells [38].

RNA binding proteins are a specific class of proteins which can regulate the formation and function of mRNAs through binding to ACUAA motifs in 3′UTR region [39]. HuR is a well-studied RNA binding protein which can adhere to *PABPN1* mRNA and promote its expression. However, hsa_circ_0031288 (CircPABPN1) is able to reduce *PABPN1* expression via sequestering and suppressing HuR [27].

### Protein coding

It was reported in 1995 that synthetic circRNAs with continuous open reading frames were capable to be translated into long-repeating polypeptide chains [40]. Several recent researches indicate that endogenous circRNAs containing open reading frames are also able to encode functional proteins (Figure 1G) [41]. For example, FBXW7–185aa translated from circ-FBXW7 and SHPRH-146aa translated from circ-SHPRH are tumor suppressors in glioblastoma [28,42].

## Nontumor diseases

In accordance with the disclosure of circRNA functions, the pivotal roles of circRNA in diseases as biomarker and regulator are catching more attention (Table 2).

**Table 2.** **Circular RNAs in nontumor diseases.**

**Disease**	**CircRNA**	**Target**	**Method involved**	**Expression**	**Function**	**Ref.**
Atherosclerosis	circANRIL	PES1 protein	PCR and RT-qPCR	Decrease	Atheroprotective	[38]

	hsa_circ_0003575	–	Microarray analysis; RT-qPCR	Increase	Repress endothelial cell proliferation and angiogenesis	[43]

CAD	circ11783–2	–	Microarray analysis; RT-qPCR	Decrease	Potential biomarker for CAD combined with T2DM	[44]

MI and heart failure	circMFACR	miR-652–3p/MTP18	PCR and RT-qPCR	Increase	Promote apoptosis and MI	[45]

	MICRA	–	RT-qPCR	Decrease	Potential biomarker for risk stratification	[46]

Hypertension	hsa_circ_0005870	–	Microarray analysis; RT-qPCR	Decrease	Potential biomarker for hypertension diagnosis	[47]

Myocardial fibrosis	circ_000203	miR-26b-5p/Col1a2, CTGF	Microarray analysis; PCR; RT-qPCR	Increase	Promote fibrosis	[48]

	circ_010567	miR-141/TGF-β1	Microarray analysis; RT-qPCR	Increase	Promote fibrosis	[49]

Cardiac hypertrophy	circHRCR	miR-223/ARC	RT-qPCR	Decrease	Repress cardiac hypertrophy	[50]

Diabetes mellitus and prediabetes	hsa_circ_0054633	–	Microarray analysis; RT-qPCR	Increase	Potential biomarker for T2DM and prediabetes diagnosis	[13]

DR	circ_0005015	miR-519d-3p	Microarray analysis; RT-qPCR	Increase	Potential biomarker for DR diagnosis; promote proliferation	[51]

	circHIPK3	miR-30a-3p/VEGFC, WNT2, FZD4	RT-qPCR	Increase	Promote proliferation and vascular dysfunction	[33]

RA	circRNA_104871	–	Microarray analysis; RT-qPCR	Increase	Potential biomarker for RA diagnosis	[52]

LN	circHLA-C	miR-150	RNA-seq; RT-qPCR	Increase	Potential biomarker for LN diagnosis	[53]

CAD: Coronary artery disease; circANRIL: Circular ANRIL; CircRNA: Circular RNA; DR: Diabetic retinopathy; LN: Lupus nephritis; MI: Myocardial infarction; RA: Rheumatoid arthritis; RT-qPCR: Reverse transcription-quantitative polymerase chain reaction; T2DM: Type 2 diabetes mellitus.

### Cardiovascular diseases

#### Atherosclerosis

Atherosclerosis is a disease in which the inside of an artery narrows due to building up of plaques, and may lead to coronary artery disease (CAD), stroke and peripheral artery disease. In a 2016 research conducted by Holdt *et al*., the atheroprotective function of circANRIL was investigated. Forced expression of circANRIL could increase cell apoptosis and decrease proliferation *in vitro* [38]. Instead of sponging miRNAs [11] or cis-regulating parental genes [6], circANRIL implemented its protective function through binding to PES1 protein, which finally resulted in the impaired ribosome maturation as well as apoptosis in smooth muscle cells and macrophages [38].

In a more recent study, Li *et al*. constructed an oxLDL-treated endothelial cell injury model in order to find potential diagnostic biomarker and elucidate molecular mechanism for atherosclerosis. They found that hsa_circ_0003575 was significantly upregulated in oxLDL-treated endothelial cells. Hsa_circ_0003575 silencing could promote cell proliferation and angiogenesis in ox-LDL-treated endothelial cells [43].

#### Coronary artery disease

CAD, also named coronary atherosclerotic heart disease, is caused by coronary artery atherosclerotic stenosis or occlusion and the sequential myocardial ischemia and hypoxia.

In a current study, Pan *et al*. investigated circRNAs in three pairs of plasma samples from CAD patients and control subjects. 24 different circRNAs were identified, and in the network constructed by bioinformatic technology, nine circRNAs could together promote *TRPM3* expression by inhibiting hsa-miR-130a-3p [19]. Although this research provides a new insight into pathological regulation of CAD, the interactions in this network are mainly based on bioinformatics analysis, and demand further investigation.

CAD is closely related with Type 2 diabetes mellitus (T2DM), and the metabolism abnormalities in T2DM directly increase CAD risk [54]. By using microarray analysis, Li *et al*. explored circRNA expression profile in CAD patients with hyperglycemia. Logistic regression analysis of two independent cohorts showed that hsa-circ11783–2 was more correlated with both CAD and T2DM than other selected circRNAs [44]. This is the first study to examine the association of circRNAs with both CAD and T2DM, and the function of hsa-circRNA11783–2 requires further exploration.

#### Myocardial infarction & heart failure

Most myocardial infarction (MI) occurs on the bases of CAD, when blood supply suddenly decreases or stops to a part of the heart. Severe MI causes cardiomyocyte death and may provoke heart failure.

In a study concerning the pathological mechanisms of MI, the authors discovered that circRNA MFACR regulated cardiomyocyte death via circMFACR/miR-652–3p/MTP18 axis [45]. MTP18 was able to induce mitochondrial fission [55] and promote cardiomyocyte apoptosis in MI [45]. MiR-652–3p was capable of inhibiting MTP18 expression and was negatively regulated by circMFACR. CircRNA MFACR elevated apoptotic cell death in MI through eliminating miR-652–3p suppression on MTP18. This research sheds new light on understanding molecular mechanism of MI [45].

A large percentage of acute MI (AMI) patients develop into heart failure due to maladaptive left ventricular remodeling. Salgado-Somoza *et al*. identified circRNA MICRA as a prognostic biomarker to improve the risk stratification after AMI. Patients with decreased MICRA level were more likely to be classified into reduced ejection fraction group. Both ordinal regression analysis and bootstrap internal validation were utilized to demonstrate the value of MICRA in prognostic stratification of AMI induced heart failure [46].

#### Hypertension

In a research published in 2016, Wu *et al*. identified circRNA expression profiles in hypertension patients’ peripheral blood. Hsa_circ_0005870 showed a significant down expression in hypertension group. Both GO and KEGG pathway analysis indicated the involvement of hsa_circ_0005870 in hypertension [47]. Hsa_circ_0005870 may represent a novel biomarker for the diagnosis of hypertension. However, the exact regulation mechanism of hsa_circ_0005870 needs further investigation.

In another study, Cheng *et al*. investigated characteristic profile of circRNA in kidney samples from four kinds of hypertension rat models. Aberrant circRNAs were identified and verified with RT-qPCR. Bioinformatics technologies were used to predict the circRNA/miRNA/mRNA network [56]. This study serves as a primary foundation for further researches concerning hypertension combined with kidney diseases.

#### Cardiomyopathy

As a kind of cardiomyopathy, the pathological progression of myocardial fibrosis is characterized with the activation of cardiac fibroblasts (CFs), in which fibroblasts transform into myofibroblasts, resulting in collagen deposition in extracellular matrix [57]. Myocardial fibrosis can be caused by deformity of cardiomyocytes resulting from metabolism disorders in diabetes mellitus patients. In two recent researches concerning circRNAs in myocardial fibrosis, both diabetic mouse and mice CFs were used as research models. CircRNA circRNA_000203 [48] and circRNA_010567 [49] were found to be remarkably upregulated in diabetic mouse myocardium and Ang-II-treated CFs. Elevated level of circRNA_000203 and circRNA_010567 accelerated fibrosis-associated protein expression. For mechanism, circRNA_000203 was demonstrated to inhibit miR-26b-5p and eliminate miRNA suppressive effect on Col1a2 and CTGF [48]. Another circRNA, circRNA_010567, was proved to sponge miR-141 and increase the expression level of TGF-β1. [49]. These two studies shed new light on the pro-fibrosis effect of circRNA_000203 and circRNA_010567, and the regulatory function of circRNA/miRNA/mRNA axis in myocardial fibrosis.

Cardiac hypertrophy is characterized by maladaptive thickening of the myocardium. Wang *et al*. revealed the circHRCR/miR-223/ARC regulatory axis in cardiac hypertrophy, which might finally develop into abnormal cardiac remodeling and heart failure. MiR-223 was capable to induce heart failure *in vivo* and cardiomyocyte hypertrophy *in vitro* [50]. ARC, a reported protein involved in pathological hypertrophy inhibition [58], was demonstrated to be the downstream target of miR-223. In order to find the antihypertrophy molecule, the authors selected 100 published circRNAs from online databases. Among them, circHRCR was significantly downregulated in pathological conditions. Further investigation demonstrated that circHRCR could repress abnormal cardiac hypertrophy and heart failure through the circHRCR/miR-223/ARC axis [50].

### Diabetes mellitus

#### Diabetes mellitus

Diabetes mellitus is a kind of metabolic disorder in which patients are affected by hyperglycemia due to inadequate insulin or insulin resistance. Diabetes mellitus can be divided into Type 1 diabetes mellitus and T2DM.

In 2016, Zhao *et al*. delineated the expression profile of circRNAs in T2DM and prediabetes patients’ peripheral blood for the first time. They selected five circRNAs as candidate biomarkers and verified in two independent cohorts. The results showed that hsa_circ_0054633 presented the highest diagnostic ability among the chosen circRNAs [13]. This study provides an insight into novel biomarker for prediabetes and T2DM.

Long-term diabetes mellitus often leads to vascular complications, including microvascular and macrovascular diseases, which are the major causes for morbidity and mortality in diabetes mellitus. CircRNAs are involved in diabetes mellitus correlated vasculopathy. For example, circWDR77 is upregulated in high glucose treated vascular smooth muscle cells. CircWDR77 regulates vascular smooth muscle cells proliferation and migration via directly binding to miR-124 and alleviating suppression for target FGF-2 [59].

#### Diabetic retinopathy

DR is one of the common complications caused by diabetes mellitus. In 2017, Gu *et al*. analyzed the altered circRNA profiles in DR patients’ serum. This is the first circRNA study concerning DR and lays the first stone for later biomarker detection and mechanism elucidation [60]. In a following study, Zhang *et al*. revealed the distinctive expression profile of circRNAs in diabetic retinas. Circ_0005015 expression was significantly upregulated in retina samples, vitreous samples, peripheral plasma samples and fibrovascular membranes of DR patients. Circ_0005015 silencing reduced human retinal vascular endothelial cells (HRVECs) proliferation, migration and tube formation. Luciferase activity assays found miR-519d-3p as the direct target for circ_0005015 [51]. Circ_0005015 is manifested as a regulatory biomarker for the DR diagnosis and treatment. In another research, Shan *et al*. demonstrated that circHIPK3 was elevated in hyperglycemia treated retinal endothelial cells and diabetic mouse retinas. CircHIPK3 served as a miRNA sponge to block miR-30a-3p activity and thus induced increase in levels of VEGFC, WNT2 and FZD4 [33]. Increment of VEGFC, WNT2 and FZD4 was reported in various retina disorders [61,62]. CircHIPK3 silencing could alleviate diabetes-induced endothelial proliferation and retina microvascular dysfunction [33].

#### Regulate insulin secretion

Several reports manifest that circRNAs are involved in the regulation of islet cells vitality. For example, Cdr1as, perhaps the best-identified endogenous mammalian circRNA, can be increased in islet cells by long-term forskolin and PMA stimulation [63]. As an miR-7 sponge [30], Cdr1as is able to improve insulin secretion and transcription through inhibiting miR-7 and accelerating *Myrip* and *Pax6* expression [63]. In another study, the authors analyzed circRNAs in human islets and cognate ones in mouse islets. They revealed that Cdr1as and circHIPK3 were abundant in normal islets, but declined in diabetic mouse. Cdr1as and circHIPK3 silencing in wild-type animal models caused defective insulin secretion and diminished islet cell proliferation. While Cdr1as performed such regulatory function by blocking miR-7, circHIPK3 regulated islet cell function by sequestering miR-124–3p and miR-338–3p and elevating *Slc2a2*, *Akt1* and *Mtpn* [64].

### Autoimmune diseases

#### Rheumatoid arthritis

Zheng *et al*. [65] and Ouyang *et al*. [52] screened the expression profile of circRNAs in rheumatoid arthritis (RA) patients’ peripheral mononuclear cells by microarray analysis. The assay results were verified by RT-qPCR method. Zheng *et al*. predicted the circRNA/miRNA interaction utilizing bioinformatic software [65]. Ouyang *et al*. analyzed the correlation between differential circRNAs and clinicopathological factors, finding that circRNA_104871 exhibited the largest diagnostic ability [52].

#### System lupus erythematosus

Li *et al*. screened circRNA profiles in system lupus erythematosus patients’ peripheral blood plasma. CircRNA candidates were selected and validated. Potential circRNA/miRNA interaction networks were constructed [66]. In another study, Luan *et al*. determined circRNA profiles in renal samples from lupus nephritis patients and health controls [53]. In their preceding investigation, the authors noticed that miR-150 was positively correlated with renal chronicity scores [67]. In the current study, they spotted circHLA-C as a probable regulator for miR-150. CircHLA-C and miR-150 exhibited a negative correlation. As a potential biomarker, circHLA-C was positively correlated with clinical factors, such as serum creatinine, renal activity index, proteinuria and crescentic glomeruli [53].

## Cancers

As it has been revealed by numerous studies, circRNAs are involved in the initiation and progression of various human cancers and may become potential diagnostic biomarkers, as it is shown in Table 3.

**Table 3.** **Circular RNAs in cancers.**

**Disease**	**CircRNA**	**Target**	**Method involved**	**Expression**	**Function**	**Ref.**
Lung cancer	hsa_circ_000064	–	RT-qPCR	Increase	Potential biomarker for lung cancer diagnosis; promote proliferation and invasion	[68]

	circRNA_100876	–	RT-qPCR	Increase	Potential biomarker for NSCLC diagnosis	[69]

	hsa_circ_0014130	–	Microarray analysis; RT-qPCR	Increase	Potential biomarker for NSCLC diagnosis	[70]

	hsa_circ_0013958	miR-134/CCND1	Microarray analysis; RT-qPCR	Increase	Potential biomarker for LAC diagnosis; promote proliferation and metastasis	[71]

	hsa_circ_0012673	miR-22/ErbB3	RT-qPCR	Increase	Potential biomarker for LAC diagnosis; promote proliferation	[72]

Breast cancer	circRNA-000911	miR-449a/Notch1	Microarray analysis; RT-qPCR	Decrease	Promote apoptosis	[73]

	circ-ABCB10	miR-1271	Microarray analysis; RT-qPCR	Increase	Promote proliferation	[74]

	hsa_circ_0001982	miR-143	Microarray analysis; RT-qPCR	Increase	Promote proliferation	[75]

	circGFRA1	miR-34a	Microarray analysis; RT-qPCR	Increase	Promote proliferation	[76]

GC	hsa_circ_0074362	–	RT-qPCR	Decrease	Potential biomarker for GC diagnosis	[77]

	hsa_circ_002059	–	RT-qPCR	Decrease	Potential biomarker for GC diagnosis	[78]

	hsa_circ_0014717	–	Microarray analysis; RT-qPCR	Decrease	Potential biomarker for GC diagnosis	[79]

	hsa_circ_0000190	–	RT-qPCR	Decrease	Potential biomarker for GC diagnosis	[80]

	hsa_circ_0000520	–	Microarray analysis; RT-qPCR	Decrease	Potential biomarker for GC diagnosis	[81]

	hsa_circ_0001017 and hsa_circ_0061276	–	Microarray analysis; RT-qPCR; RT-ddPCR	Decrease	Potential biomarker for GC diagnosis	[82]

	hsa_circ_100269	miR-630	RT-qPCR	Decrease	Potential biomarker for GC diagnosis; repress proliferation	[83]

	circLARP4	miR-424–5p/LATS1	Microarray analysis; RT-qPCR	Decrease	Potential biomarker for GC prognosis; repress proliferation and invasion	[84]

HCC	hsa_circ_0001649	–	RT-qPCR	Decrease	Potential biomarker for HCC diagnosis	[85]

	hsa_circ_0005075	–	Microarray analysis; RT-qPCR	Increase	Potential biomarker for HCC diagnosis	[86]

	circZKSCAN1	–	RT-qPCR	Decrease	Potential biomarker for HCC diagnosis; repress proliferation, migration and invasion	[87]

	circMTO1	miR-9/p21	Microarray analysis; RT-qPCR	Decrease	Potential biomarker for HCC prognosis; repress proliferation and invasion	[88]

	cSMARCA5	miR-17–3p, miR-181b-5p/TIMP3	RNA-seq; RT-qPCR	Decrease	Potential biomarker for HCC prognosis; repress proliferation and migration	[89]

	circ_0067934	miR-1324/FZD5	RT-qPCR	Increase	Promote proliferation and migration	[90]

	circHIPK3	miR-124/AQP3	RT-qPCR	Increase	Promote proliferation and migration	[32]

	circRNA_100338	miR-141–3p	Microarray analysis; RT-qPCR	Increase	Promote migration and invasion	[91]

CRC	circRNA0003906	–	RT-qPCR	Decrease	Potential biomarker for CRC diagnosis	[92]

	hsa_circ_001988	–	RT-qPCR	Decrease	Potential biomarker for CRC diagnosis	[93]

	hsa_circ_103809 and hsa_circ_104700	–	Microarray analysis; RT-qPCR	Decrease	Potential biomarker for CRC diagnosis	[94]

	hsa_circ_0000069	–	Microarray analysis; RT-qPCR	Increase	Potential biomarker for CRC diagnosis; promote proliferation, migration and invasion	[95]

	hsa_circ_0007534	–	Microarray analysis; RT-qPCR	Increase	Potential biomarker for CRC diagnosis; promote proliferation	[96]

	hsa_circ_0020397	miR-138/TERT, PD-L1	RT-qPCR	Increase	Potential biomarker for CRC diagnosis; promote invasion	[97]

Bladder cancer	circTCF25	miR-103a-3p, miR-107/CDK6	Microarray analysis; RT-qPCR	Increase	Promote proliferation and migration	[98]

	circRNA-MYLK	miR-29a/VEGFA	Microarray analysis; RT-qPCR	Increase	Promote cancer proliferation	[99]

	circBCRC4	miR-101/EZH2	RT-qPCR	Decrease	Promote apoptosis	[100]

	circ-ITCH	miR-17, miR-224/p21, PTEN	RT-qPCR	Decrease	Repress proliferation and migration	[101]

Glioma	hsa_circ_0046701	miR-142–3p/ITGB8	RT-qPCR	Increase	Promote proliferation and invasion	[102]

	circ-FBXW7	–	RNA-seq; RT-qPCR	Decrease	Code protein	[28]

	circ-SHPRH	–	RNA-seq; RT-qPCR	Decrease	Code protein	[42]

CircRNA: Circular RNA; CRC: Colorectal cancer; GC: Gastric cancer; HCC: Hepatocellular carcinoma; LAC: Lung adenocarcinoma; NSCLC: Non-small-cell lung cancer; RT-ddPCR: Reverse transcription-droplet digital polymerase chain reaction; RT-qPCR: Reverse transcription-quantitative polymerase chain reaction.

### Lung cancer

Lung cancer, also known as lung carcinoma, is the leading cause of cancer death globally [103]. According to histopathological classification, lung cancer can generally be divided into non-small-cell lung cancer (NSCLC) and small cell lung cancer.

Luo *et al*. reported that hsa_circ_0000064 exhibited elevated expression in both lung cancer tissues and lung cancer cell lines (A549 and H1229). There was a close correlation between hsa_circ_0000064 augmentation and tumor differentiation, tumor-lymph node-metastasis (TNM) stage and lymphatic metastasis. Hsa_circ_0000064 silencing suppressed the proliferation and migration of cancer cells, and promoted cell apoptosis. For molecular mechanisms, the authors discovered that hsa_circ_0000064 regulated apoptotic-related proteins, cycle-related proteins and invasion-related proteins [68].

NSCLC accounts for nearly 85% of all primary lung cancers. Yao *et al*. revealed that circRNA_100876 was elevated in NSCLC tissues compared with pair-matched nontumor tissues. The upregulated expression was correlated with TNM stage and lymphatic metastasis. Furthermore, NSCLC patients with higher circRNA_100876 level had an overall shorter survival time than NSCLC patients with lower expression level [69]. In another study, hsa_circ_0014130 was found to be notably upregulated in NSCLC tissues and the expression was also associated with both TNM stage and lymphatic metastasis. The AUC was 0.878. Then, the circRNA/miRNA interaction network was predicted by bioinformatics technologies [70].

Lung adenocarcinoma (LAC) is currently the most common subtype of NSCLC in lifelong nonsmokers [104]. Zhao *et al*. investigated circRNA profile in early-stage LAC patients’ tumor tissue and adjacent normal tissue. Five dysregulated circRNAs were validated and potential circRNA/miRNA network was predicted [105]. In another study, hsa_circ_0013958 was detected as a LAC biomarker with regulatory potency. Hsa_circ_0013958 was increased in LAC cell lines, cancer tissues and cancer patients’ plasma. The expression level of hsa_circ_0013958 was associated with tumor staging and lymph node metastasis and the AUC was 0.815. Biological function experiments validated that hsa_circ_0013958 was involved in cellular proliferation and metastasis. Finally, the hsa_circ_0013958/miR-134/CCND1 regulatory axis was constructed [71]. Similarly, the expression level of hsa_circ_0012673 was significant overexpressed in LAC tissues, and was associated with tumor size. Hsa_circ_0012673 could accelerate LAC proliferation through sequestering miR-22, resulting in elevated level of targeted ErbB3 [72].

### Breast cancer

The most commonly diagnosed cancer among women has been breast cancer in recent years, which is also the leading cause of cancer death in women younger than 45 years [106]. In a present study, a circRNA with antioncogenic role was found in breast cancer. CircRNA-000911 was significantly decreased in breast cancer tissues and cancer cell lines. Overexpression of circRNA-000911 could induce decreased cell proliferation, migration and invision as well as increased apoptosis. CircRNA-000911 implemented its regulatory function via inhibiting miR-449a and promoting Notch1 and NF-κB signaling pathway [73]. In the study performed by Liang *et al*., the authors revealed that circ-ABCB10 was significantly increased in breast cancer tissues. SiRNA induced circ-ABCB10 silencing contributed to decreased cancer cell proliferation and elevated apoptosis. Function analysis demonstrated that circ-ABCB10 carried out its tumorigenesis role via binding to miR-1271 [74]. Similar tumor-promoting effect was also observed in hsa_circ_0001982 targeting miR-143 [75].

Triple negative breast cancer is a kind of breast cancer not sensitive to hormone therapies targeting ER, PR or Her2/neu. He *et al*. analyzed circRNA patterns in cancer cell lines and verified one upregulated circRNA circGFRA1. Silencing of circGFRA1 relieved suppression for target miR-34a, which results in inhibited cancer cell proliferation and promoted apoptosis [76]. Yan *et al*. revealed that circVRK1 was reduced in breast cancer stem cells. CircVRK1 repressed stemness-maintenance ability of breast cancer stem cells [107]. Furthermore, miR-153–5p, the predicted target of circVRK1, was previously reported to be involved in stemness-maintenance of triple negative breast cancer [108].

### Gastric cancer

Gastric cancer (GC) is among the leading causes of cancer death in developing countries nowadays [109]. Several researches delineated the global expression patterns of circRNAs in GC tissues or patients’ plasma [79,82,110,111]. Part of the results was validated with RT-qPCR. These investigations lay the foundation for future exploration in GC.

From 2015 to 2017, quite a few circRNAs were detected as potential GC biomarkers, such as hsa_circ_0074362 [77], hsa_circ_002059 [78] and hsa_circ_0014717 [79]. All of these diagnostic biomarkers were dysregulated in cancer tissue compared with paired noncancerous tissue and were correlated with several clinical-pathological factors. For example, Hsa_circ_0074362 was significantly down-expressed in GC tissues, cancer cell lines and gastritis. The level of hsa_circ_0074362 in GC was significantly lower compared with moderate gastritis. The expression level of hsa_circ_0074362 was associated with CA19–9 and lymphatic metastasis. The receiver operator curve was 0.630 [77]. These suggest that hsa_circ_0074362 may have potential values in the screening of GC.

Some circRNAs as biomarkers not only exist in tumor tissues, but also exist in patients’ plasma, such as hsa_circ_0000190 [80], hsa_circ_0000520 [81], hsa_circ_0001017 and hsa_circ_0061276 [82]. For instance, Li *et al*. demonstrated that hsa_circ_0001017 and hsa_circ_0061276 expression levels were downregulated in both cancer tissues and paired plasma and were associated with tumor size and distal metastasis. Through combining the expression levels of hsa_circ_0001017 and hsa_circ_0061276 in tissue and plasma, the AUC could ascend to 0.966 [82].

Among these disclosed circular biomarkers, a few are manifested as functional miRNA sponges. In their previous study, Zhang *et al*. noticed the remarkable decline of hsa_circRNA_100269 in recurrent GC tissue [112]. In their follow-up examination, they manifested that hsa_circRNA_100269 overexpression could prohibit tumor cell proliferation via absorbing miR-630 [83]. Another example of regulatory circRNAs in GC is circLARP4. Based on their preceding research concerning mRNA LATS1 as a tumor suppressor in GC [84], Zhang *et al*. explored the upstream regulatory network for LATS1. They revealed that circLARP4 could impede GC cell proliferation and invasion by targeting miR-424–5p, which led to raised LATS1. However, only early stage patients with a higher circLARP4 expression had a better outcome than patients with circLARP4 low expression. Similar trends could not be observed in late stage patients [113].

### Hepatocellular carcinoma

HCC is the most common type of primary liver cancer and the third leading cause of cancer mortality in many countries [114]. Early researches demonstrated diagnostic potency of circRNAs in HCC. For instance, both hsa_circ_0001649 and hsa_circ_0005075 were identified as novel biomarkers for HCC [85,86]. Later researches concern not only about diagnostic ability but also regulatory potency of circRNAs in HCC. Some circRNAs display antioncogenic effects. Yao discovered that, in HCC tissue samples, both circZKSCAN1 and ZKSCAN1 mRNA were significantly lower compared with noncancer tissues. Blocking circZKSCAN1 and/or ZKSCAN1 mRNA would promote cancer cell proliferation, migration and invasion. Furthermore, circZKSCAN1 was associated with tumor numbers, cirrhosis, vascular invasion and tumor grade [87]. Another two circRNAs, circMTO1 and cSMARCA5, were also downregulated in HCC tissue. Both of them could inhibit HCC cell proliferation and migration. CircMTO1 exerted tumor suppressive role via circMTO1/miR-9/p21 axis [88], and cSMARCA5 via cSMARCA5/miR-17–3p, miR-181b-5p/TIMP3 axis [89]. Some circRNAs act as tumor-promoting molecules in HCC. Circ_0067934 and circHIPK3 were all highly expressed in HCC tissues. Knockdown of these circRNAs resulted in suppressed HCC cell proliferation and migration [32,90]. Circ_0067934 achieved its regulatory function through circ_0067934/miR-1324/FZD5 pathway [90], and circHIPK3 through circHIPK3/miR-124/AQP3 axis [32].

A majority of HCC in Asian area arises from chronic hepatocellular B virus (HBV) infection and subsequent cirrhosis [115]. Cui *et al*. clarified circRNA expression profiles in HBV-related HCC by microarray analysis [116]. Huang *et al*. discovered that circRNA_100338 was upregulated in HBV-related HCC, and correlated with both low survival rate of patients and invasive process of cancer cells. CircRNA_100338 could facilitate cancer cell invasion and migration by sponging and inhibiting miR-141–3p [91].

### Colorectal cancer

In recent years, several circRNAs are detected as candidate biomarkers for CRC. For instance, the expression level of circRNA0003906 was significantly decreased in CRC tissues and CRC cell lines, and was correlated with clinicopathological factors, such as lymphatic metastasis and poor differentiation. The AUC was 0.818, which demonstrated the diagnostic ability of circRNA0003906 [92]. Similarly, hsa_circ_001988 [93], hsa_circRNA_103809 and hsa_circRNA_104700 [94] were also demonstrated as potential diagnostic biomarkers. Their expression levels were dysregulated in CRC and correlated with clinicopathological features. The AUC for hsa_circ_001988, hsa_circRNA_103809 and hsa_circ_104700 was 0.788, 0.699 and 0.616, respectively [93,94].

Several ectopic circRNAs were verified to have regulatory potential in CRC. Gua *et al*. revealed that hsa_circ_0000069 silencing notably attenuated tumor cell proliferation, migration and invasion [95]. Zhang *et al*. testified that hsa_circ_0007534 silencing led to suppressed proliferation and induced apoptosis of CRC cells [96]. In another research, the authors delineated that hsa_circ_0020397 performed its tumor-generating effects via hsa_circ_0020397/miR-138/TERT, PD-L1 axis [97].

Metastasis is among the major causes of tumor death and often occurs in late stages of tumor development [117]. Two recent studies investigated the metastasis in CRC by utilizing circRNA profiling. Jiang *et al*. detected differential circRNAs in CRC metastasis cells [118], and Zeng *et al*. compared circRNA expression in CRC patients with and without lung metastasis [119]. These findings provided candidate circRNAs for later investigations concerning CRC metastasis.

### Bladder cancer

Recent studies reveal certain circRNAs with regulatory function in bladder cancer. Zhong *et al*. discovered that forced expression of circTCF25 would sequester miR-103a-3p and miR-107, which led to the increased CDK6 expression and promoted cancer cell proliferation and migration [98]. In another study, the authors manifested that circRNA-MYLK and its downstream target miR-29a/VEGFA, VEGFR2 signaling pathway were related with bladder cancer cell proliferation and epithelial–mesenchymal transition process [99]. Li *et al*. discovered that increased expression of circRNA BCRC4 could induce bladder cancer cell apoptosis by inhibiting miR-101 ability, which relieved suppression for EZH2 [100].

Circ-ITCH is generated from itchy E3 ubiquitin protein ligase (*ITCH*) coding region and is involved in bladder cancer. For instance, Yang *et al*. found that circ-ITCH was downregulated in bladder cancer tissues and cell lines. Decreased expression of circ-ITCH was correlated with shortened survival in bladder cancer patients. Expression of circ-ITCH represses cancer cell proliferation, migration and metastasis. This consequence of circ-ITCH was achieved by inhibiting miR-17 and miR-224 and promoting p21 and PTEN [101].

### Glioma

In 2016, Song *et al*. constructed a computational filter named UROBORUS and disclosed circRNA expression in gliomas for the first time [120]. Then Li *et al*. revealed that hsa_circ_0046701 was increased in glioma tissue and cell lines. Hsa_circ_0046701 was able to regulate tumor cell proliferation and invasion by sponging miR-142–3p and increasing ITGB8 [102]. Interestingly, the open reading frame equipped in circ-FBXW7 and circ-SHPRH allowed them to translate functional proteins, FBXW7–185aa and SHPRH-146aa [28,42]. Both FBXW7–185aa and SHPRH-146aa displayed antioncogenic potency in glioma. These two researches indicated a potential new role for circRNAs.

## Conclusion & future perspective

Unlike linear RNAs, continuous circRNAs have no free ends and are more resistant to RNase. The properties of circRNAs include stability, prevalence, specificity and conservation. CircRNAs have been demonstrated to regulate cellular function through sponging other factors (miRNAs or proteins), promoting transcription or coding proteins. From cancers to noncancerous disorders, large quantities of studies have revealed the involvement of circRNAs in human diseases as biomarkers and/or regulators. These potential circular biomarkers/regulators, combined with currently widely used diagnostic and treating methods, may improve future clinical activities. However, in order to contribute to later diagnosis and treatment, the precise role of circRNA in both physiological and pathological conditions needs further investigation. Besides the reported mechanism, new hypothesis may be proposed and demonstrated. What is more, the curative effects and the side effects of circRNAs as treatment targets *in vivo* should be evaluated and examined.

**Executive summary**

- Circular RNAs (circRNAs) are generated without 3′ and 5′ free ends.
- CircRNAs include exonic RNAs, exon–intron RNAs and circular intronic RNAs.
- CircRNAs are stably, prevalently, specifically and conservatively expressed.
- CircRNAs regulate gene expression and transcription through binding to miRNAs, Pol II and proteins. Some endogenous circRNAs code proteins themselves.
- CircRNAs are related with diverse human diseases.
